# Musashi-2 (MSI2) regulation of DNA damage response in lung cancer

**DOI:** 10.21203/rs.3.rs-4021568/v1

**Published:** 2024-04-11

**Authors:** Igor Bychkov, Alexander Deneka, Iuliia Topchu, Ragendra Pangeni, Amr Ismail, Christopher Lengner, John Karanicolas, Erica Golemis, Peter Makhov, Yanis Boumber

**Affiliations:** Fox Chase Cancer Center; Fox Chase Cancer Center; Northwestern University; Nova Southeastern University; University of Alabama in Birmingham; University of Pennsylvania; Fox-Chase Cancer Center; Fox Chase Cancer Center; Fox Chase Cancer Center; University of Alabama in Birmingham

## Abstract

Lung cancer is one of the most common types of cancer worldwide. Non-small cell lung cancer (NSCLC), typically caused by *KRAS* and *TP53* driver mutations, represents the majority of all new lung cancer diagnoses. Overexpression of the RNA-binding protein (RBP) Musashi-2 (MSI2) has been associated with NSCLC progression. To investigate the role of MSI2 in NSCLC development, we compared the tumorigenesis in mice with lung-specific *Kras*-activating mutation and *Trp53* deletion, with and without *Msi2* deletion (KPM2 versus KP mice). KPM2 mice showed decreased lung tumorigenesis in comparison with KP mice. In addition, KPM2 lung tumors showed evidence of decreased proliferation, but increased DNA damage, marked by increased levels of phH2AX (S139) and phCHK1 (S345), but decreased total and activated ATM. Using cell lines from KP and KPM2 tumors, and human NSCLC cell lines, we found that MSI2 directly binds *ATM* mRNA and regulates its translation. MSI2 depletion impaired DNA damage response (DDR) signaling and sensitized human and murine NSCLC cells to treatment with PARP inhibitors *in vitro* and *in vivo*. Taken together, we conclude that MSI2 supports NSCLC tumorigenesis, in part, by supporting repair of DNA damage by controlling expression of DDR proteins. These results suggest that targeting MSI2 may be a promising strategy for lung cancers treated with DNA-damaging agents.

## Introduction

Lung cancer is one of the most frequently diagnosed cancers and is the leading cause of cancer related death worldwide. Based on World Health Organization data [1], lung cancer counts for > 2 million (11.4%) of new cases and 1.8 million (18%) of lung cancer deaths in 2020. Owing to the absence of clinical symptoms in early-stage disease and limitations of effective screening programs, most lung cancers are diagnosed in advanced stages. The most common type of lung cancer is non-small cell lung cancer (NSCLC), representing 85% of total lung cancer cases [2], which includes adenocarcinoma, squamous cell carcinoma and large-cell carcinoma histologic subtypes. Recent cancer genome sequencing efforts have defined the complex of genomic aberrations that lead to lung cancer development [3]. In NSCLC, druggable alterations include mutations in Epidermal Growth Factor Receptor (EGFR), ALK and ROS rearrangements and others, and occur in ~ 10–60% of tumors [4]. Approximately 30% of lung adenocarcinomas are driven by KRAS mutations [5], some of which are druggable. However, many NSCLC do not respond well to treatment, and overall survival for NSCLC patients diagnosed at a late stage remains 26% [6].

*KRAS* mutations often co-exist with mutations impairing activity of the *TP53* tumor suppressor, contributing to increased genomic damage [7]. The DNA damage response (DDR) signaling network regulates activation of transcription, cell cycle, apoptosis, senescence, and DNA repair processes in response to DNA damage [8]. Coordination of these biological processes is critical for cell survival when DNA replication is perturbed or when cells are treated with mutation-inducing agents. A pair of related protein kinases, Ataxia-telangiectasia mutated (ATM) and Ataxia-telangiectasia and Rad3-related protein (ATR), are activated by DNA damage. ATM is regulated by the MRN (Mre11-Rad50-NBS1) complex, which senses double-strand breaks (DSBs), whereas ATR is regulated by ATRIP (ATR-interacting protein), which senses single-strand DNA (ssDNA) generated by processing of DSBs, as well as ssDNA present at stalled replication forks [9–12]. The poly (ADP-ribose) polymerase (PARP) protein is critical for several forms of DNA repair, including nucleotide excision and base excision repair (NER and BER) and homologous recombination (HR), and supports repair of DNA damage induced by alkylating agents and chemotherapy. Several recent clinical studies have recently shown potential efficacy of PARP inhibitors in NSCLC, especially when combined with platinum-based chemotherapy or immunotherapy, although there is lack of clarity of specific biomarkers for patient selection that benefit from this approach [13–16]. Interestingly, up to 40% of human lung adenocarcinomas lack ATM protein expression, implicating altered DDR in a subset of NSCLC [17]. This provides the basis for a potentially therapeutic approach, as recent publications show that ATM deficiency in NSCLC is associated with higher sensitivity to the PARP inhibitor olaparib and the ATR inhibitor AZD6738 [18–20].

Our previous work has shown the elevated expression of MSI2 in advanced NSCLC [21, 22]. MSI2 is an RNA-binding protein which regulates the stability and translation of target mRNAs via recognition of specific core motifs in the 3’-untranslated region (UTR) [23–25]. MSI2 and its homolog, MSI1, regulate multiple critical biological processes relevant to stem cell compartment maintenance, cancer progression, and cancer drug resistance [26, 27], and have elevated expression in numerous types of cancer [21, 24, 28–31]. In studies of cell lines established from highly metastatic tumors arising in mice expressing lung-specific activated KRAS and TP53 deletion (129S/*Sv-Kras*^*tm3Tyj*^/J;*Trp53*^*t*m1Brn^/J (KP) mice), we previously established MSI2 as upregulated in a subset of aggressive NSCLC and demonstrated a specific role for MSI2 in promoting metastasis in these tumors, through induction of TGFβR1 and its effector SMAD3 [22]. In other studies, using human cell models, we have shown that MSI2 directly binds to EGFR mRNA and regulates EGFR protein expression, while depletion of MSI2 has led to increased sensitivity to EGFR inhibitors in EGFR-mutant NSCLC [21]. Also, we have shown that MSI2 directly regulates PTEN and VEGFR2 protein levels in NSCLC [32]. These data suggested a broad role for MSI2 in increasing tumor aggressiveness.

In this study, we analyzed the effect of simultaneous deletion of *Msi2* [33] on the pathogenesis and therapeutic response of KP mice [34], creating and analyzing a new (*Kras*^*mut*^/*P53*^*KO*^/*MSI2*^*KO*^ or KPM2 model. The new murine KPM2 model showed decreased total tumor number and burden in comparison with positive control (KP) and lung cancer cell lines generated from those models, show decreased proliferation activity of KPM2 cells in comparison with KP. Strikingly, KPM2 tumors show significantly elevated levels of DNA breaks in contrast with KP tumors, and the KPM2 genotype is associated with significantly altered DDR signaling *in vitro* and *in vivo*. MSI2 deficiency also leads to higher sensitivity to PARP (Poly (ADP-ribose) polymerase) inhibitors and cisplatin in NSCLC models. Together, these results suggest that targeting MSI2 may be a promising adjuvant strategy for treatment of lung cancer.

## Materials and methods

### Animals.

All animal experiments were approved by the Institutional Animal Care Committees at Fox Chase Cancer Center and Robert H Lurie Comprehensive Cancer Center. To generate A novel KP-MSI2 model (129S/Sv-*Kras*^*tm3Tyj/*J^;*Trp53*^*t*m1Brn^/^J^; *Msi2*^−/−^), we crossed 129S/Sv-*Kras*^*tm3Tyj*/J^;*Trp53*^*t*m1Brn/J^ (KP) mice[34] with C57BL/6 floxed Msi2^−/−^Cre transgenic mice [33]. Following lung-specific induction with Adeno-Cre, each 6-week-old mouse get 2.5 ×10^6^ units of Cre recombinase adenovirus (Vector Biolabs, Malvern, PA), the 129S/Sv-*KP* mice develop lung disease similar to that of humans; large papillary adenomas are seen after 6 weeks and adenocarcinoma after ~ 10 weeks, in 4-month-old animals [34]. Mice were euthanized after ~ 5 month of activation and their lungs, heart, liver, spleen were collected for paraffin blocks, and lung cancer cell lines were established from fresh lung mouse tumors.

### Mouse genotyping.

Small segments of mouse tails (0.5 ~ 1.0 cm) were collected and digested in 150 μL of DNA-lysis buffer with 0.2 mg/ml of proteinase K for overnight at 56 °C. Then, DNA samples were diluted 50 times and mixed with DreamTaq master mix (2X) (Thermofisher) following vendor protocol for genotyping PCR. Then, DNA electrophoresis was performed in 2% agarose gel. Bands detection was performed using imaging system ChemiDoc (Bio-Rad). All primers used in this procedure are noted in Supp Table S1.

### Assessment of in vivo tumor growth.

For *in vivo* studies 2 × 10^6^ of KP-1 and KPM2–2 cells were injected subcutaneously (s.c.) in the right flank of 6-week-old mice C57BL/6 (total: 20 males, 20 females) from The Jackson Laboratory (ME, USA). All mice were over 18g at the start of live-phase study. When tumor volumes reached 100 mm^3^ animals were randomly assigned to the control or experimental groups (n = 10 mice/group, 5 males, 5 females). The mice were treated with 5% DMSO, 40% PEG 300, 5% Tween-80, 50% sterile H_2_O (vehicle) or olaparib 50 mg/kg in 5% DMSO, 40% PEG 300, 5% Tween-80, 50% sterile H_2_O. Treatment was perfomed by daily oral gavage. Tumors were measured twice weekly, and their volumes were calculated with the formula: [volume = 0.52 × (width)^2^ × length]. Mice were euthanized when tumor volume exceeded 1500 mm^3^ or on 41st day of experiment.

### Immunohistochemistry of mouse NSCLC samples.

Scientific review committee (SRC) and IRB approvals were obtained for de-identified samples from Fox Chase Cancer Center, both at Fox Chase Cancer Center and at Robert H Lurie Comprehensive Cancer Center. Tissue samples were stained for MSI2, MSI1, ATM and CyclinB1, Ki-67 and phospho-H2AX via immunohistochemical (IHC) approach and hematoxylin and eosin (H&E) stained sections were used for morphological evaluation purposes, and unstained sections were used for IHC staining using standard methods. Briefly, 5 μm formalin-fixed, paraffin-embedded sections were deparaffinized and hydrated. Sections were then subjected to heat-induced epitope retrieval with 0.01M citrate buffer (pH 6.0) (MSI2, MSI1, ATM) or EDTA buffer (CyclinB1, Ki-67, phospho-H2AX). Endogenous peroxidases were quenched by the immersion of the slides in 3% H_2_O_2_ solution. The sections were incubated overnight with primary antibodies at 4°C in a humidified slide chamber. As a negative control, the primary antibody was replaced with normal mouse/rabbit IgG to confirm absence of specific staining. Immunodetection was performed using the Dako Envision + polymer system and immunostaining was visualized with the chromogen 3, 3′-diaminobenzidine. All slides were viewed with a Nikon Eclipse 50i microscope and photo- micrographs were taken with an attached Nikon DS-Fi1 camera (Melville, NY, USA). For IHC quantification, each spot was examined by board-certified pathologists (ED and NK) who assigned a score of 0 (no staining), 1+ (weak staining), 2+ (moderate staining), and 3+ (strong staining) within carcinomatous areas. The score for each of the two tumor spots was averaged for statistical analysis. The H-score, which ranges from 0 to 300, was calculated using the following formula: [1x(% cells 1+) + 2x(% cells 2+) + 3x(% cells 3+)], which reflects staining intensity as well as percentage of positive cells. A sum of p2 and p3 represents a sum of 2 + and 3 + cells (2× (% cells 2+) + 3×(% cells 3+), which excludes 1 + cells

### Establishment of cell lines.

Murine lung tumors were collected and used to generate. murine NSCLC cell lines by standard approaches. Briefly, the lines 129S/Sv-*Kras*^*tm3Tyj*/J^;*Trp53*^*t*m1Brn^/^J^; *Msi2*^−/−^ (KPM2–1, KPM2–2, KPM2–3) and *Kras*^*tm3Tyj*/J^;*Trp53*^*t*m1Brn/J^ (KP-1, KP-2, KP-3) were prepared from freshly isolated cancer cells. Initial stocks were cryopreserved, and at every 6-month interval, a fresh aliquot of frozen cells was used for the experiments. The tumor pieces were incubated with collagenase (400 u/ml) at 37 °C for 2 hours, then we used cell strainer (70 μm) for cells separation. After that, the cells were incubated with ACK lysis buffer at room temperature for 1–2 minutes, then the cells were washed and transferred to T25 flask with B27 cell culture media for propagation.

### Cell culture of human cancer cell lines.

Human lung cancer cell lines with KRAS-mutations (A549 and H441) were obtained from the American Type Culture Collection (ATCC). No additional authentication was performed. All cells were cultured in RPMI 1640 (Gibco, Gaithersburg, MD) supplemented with 10% FBS (Hyclone, Logan, UT), penicillin (100U/ml), streptomycin (100μg/ml), sodium pyruvate (1 mM) and non-essential amino acids (0.1 mM) under conditions indicated in the figure legends.

### Antibodies and drugs.

Anti-MSI2 (#ab76148), anti-MSI1 (#ab21628), anti-phCHK2 T68 (#ab278548) were obtained from Abcam (Cambridge, UK). Anti-phATM S1981 (#GTX132146), anti-ATM (#GTX70103), anti-ATR (#GTX128146), anti-phCDC25A (#GTX55131), anti-CDC25A (#GTX102308) were obtained from GeneTex, Inc. (Irvine, CA). Anti-phH2A.X S139 (#9718), anti-ATM (#2873), anti-CHK2 (#2662), anti-phCHK1 S345 (#2348), anti-CHK1 (#2360), anti-β-actin (#3700), anti-rabbit HRP-linked (#7074), anti-mouse HRP-linked (#7076) were obtained from Cell signaling (Danvers, MA). Anti-MSI1 (#AF2628) was obtained from R&D systems (Minneapolis, MN) Anti-ATM (#27156–1-AP) was obtained from Proteintech (Rosemont, IL). Olaparib (#HY-10162), cisplatin (#HY-17395), doxycycline (#HY-N0565) were obtained from MedChemExpress (Monmouth Junction, NJ).

### Vector construction and lentivirus production.

To generate stable human cell lines with inducible MSI2 knockdowns, self-complementary single-stranded DNA oligos (Supp Table S2) were annealed and cloned into AgeI/EcoR1 sites of Tet-pLKO-puro vector (Addgene plasmid # 21915). All constructs were validated by direct sequencing. All generated cell lines used in the study are noted in Supp Table S3, and have been previously described [21, 32].

### Western blot analysis.

Cell lysates preparation and Western blot analysis were performed using standard methods as previously described[21]. Signals were detected by X-ray films and digitized by photo scanner. Image analysis was done using ImageJ (version 1.53e, National Institutes of Health, Bethesda, MD), with signal intensity normalized to β-actin; 3–4 repeats were used for each quantitative analysis. Final data was analyzed in GraphPad Prism by unpaired t-test to determine statistical significance.

### Reverse transcription and qPCR.

RNA was extracted using a phenol-chloroform based method. RNA concentration and quantity were measured using NanoDrop Lite (cat# ND-LITE ThermoFisher Scientific). First strand cDNA synthesis was performed with iScript cDNA synthesis kit (cat#1708841, Biorad, California, USA) according to manufacturer’s instructions. The generated cDNA was diluted tenfold and used as a template for qPCR, which was performed with Applied Biosystems QuantStudio 3 system using PowerTrack^™^ SYBR Green Master Mix (Applied Biosystems). Relative quantification of genes expression was performed using 2^−ΔΔCt^ method, Ct greater than 35 cycles was considered as undetected, using primers indicated in Supp Table S4.

### Cell viability assay.

To analyze the effects of MSI2 depletion or compound treatment on cell proliferation, cells were plated (500 cells/well) in 96-well cell culture plates in complete media. We used increasing concentrations of compounds to calculate IC_50_ values for each cell line. After 72 hours incubation with compounds, we added CellTiter-Blue^®^ reagent (Promega, Fitchburg, WI) to cells and incubated them for 2 hours at 37 °C. After that we recorded fluorescence (560_Ex_/590_Em_). Data was analyzed using GraphPad Prism software.

### Clonogenic assay.

Murine KP, KPM2 cells and and human A549 cells (500 cells per well) and H441 cells (2000 cells per well) were plated in 12-well pates and incubated in complete media. After 24 hours compounds were added, after which cells were incubated for 7 days. Cells were fixed in 10% acetic acid/10% methanol solution and stained with 0.5% (w/v) crystal violet as previously described[35]. A colony was defined as consisting of > 50 cells and counted digitally using ImageJ software as described previously[36].

### RNA-IP assays.

RNA was immunoprecipitated from cell lysates (2 × 10^7^ cells per IP) using either a control normal rabbit IgG or rabbit monoclonal anti-MSI2 antibody and the Magna RIP RNA-binding Protein Immunoprecipitation kit (cat#17–700, Millipore, Burlington, MA). The manufacturer’s instructions were followed with the exception that RNeasy MinElute Cleanup kit (cat#74202, Qiagen, Venlo, Netherlands) was used to prepare RNA. Immunoprecipitated RNAs were quantified by quantitative PCR (qPCR) using primers indicated in Supp Table S4, using PTP4A1 as a normalization (positive control) and GAPDH as a negative control.

### RPPA.

The murine cell lines were lysed and prepared according to MD Anderson Core Facility instructions[37–39], and RPPA was performed at MD Anderson facility. Heatmap was generated using the GraphPad Prism.

### In silico evaluation of MSI2 binding to mRNAs.

Human and murine genome sequences for ATM and other mRNAs from the NCBI (ATM human (NCBI Reference Sequence: NM_000051.4), ATM mouse (NCBI Reference Sequence: NM_007499.3), CHK2 human (NCBI Reference Sequence: NM_001005735.2), CHK2 mouse (NCBI Reference Sequence: NM_001363308.1) were scanned for Musashi binding motifs previously defined by Bennett *et al*. (15 motifs with highest p values) [23], Wang *et al*. (8 motifs with highest p values) [24] and Nguyen *et al*. (12 motifs with highest p values) [25] (Supp Tables S5, S6, S7).

### Statistical analysis.

All statistical analyses, including unpaired two-tailed t-test, ANOVA analysis, Spearman correlation, were performed in GraphPad Prizm 9 (San Diego, CA).

## Results

### Musashi-2 contributes to the incidence of KRAS-P53 driven mouse lung adenocarcinoma and positively regulates lung cancer cell growth.

**Figure 1:**
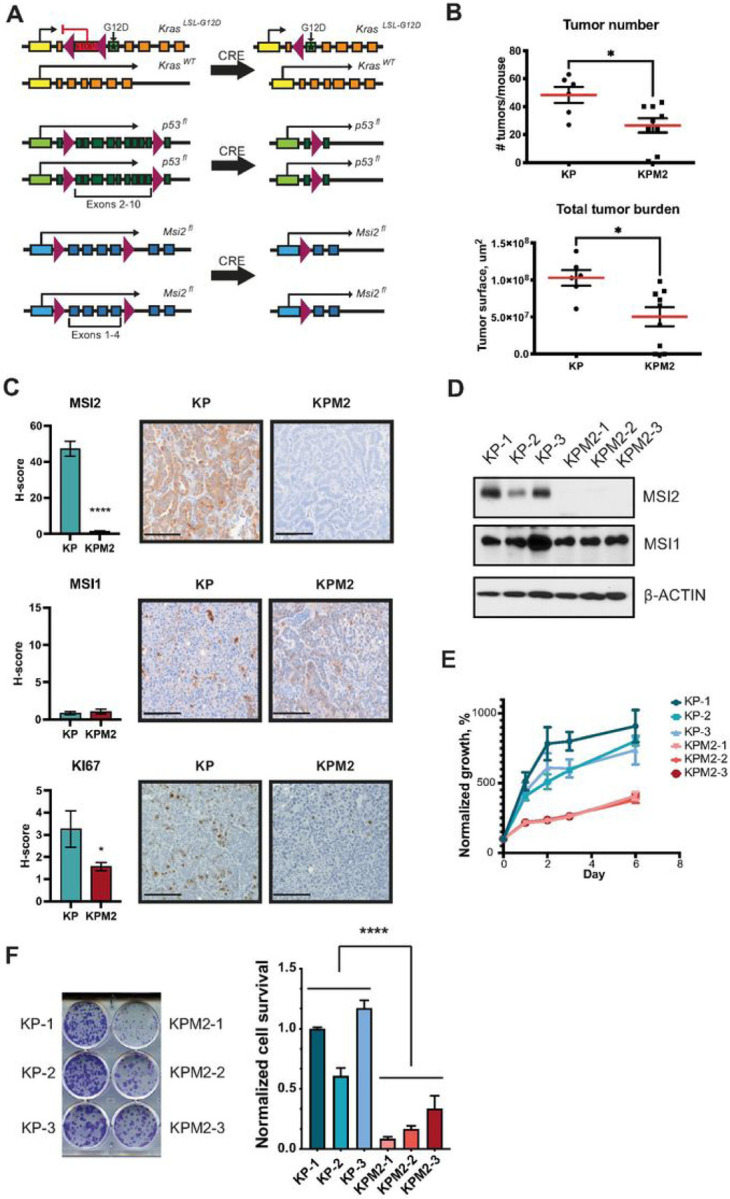
Figure legend not available with this version.

To assess the role of MSI2 in NSCLC development *in vivo*, we generated a new murine model (KPM2) by crossing 129S/Sv-*Kras*^*tm3Tyj*/J^;*Trp53*^*t*m1Brn/J^ (KP)[34] and *Msi2*^−/−^ mice [33] (Fig. 1A). In these mice, intratracheal administration of lentivirus expressing Cre recombinase simultaneously activates *Kras*^*G12D*^ and deletes P53 to initiate tumorigenesis in the lung epithelium, and for those bearing *Msi2*^−/−^, Msi2 is concurrently deleted. Induction of Cre in KP and KPM2 mice induced lung tumors in both KP and KPM2 mice after 10 weeks. KPM2 mice had significantly fewer distinguishable independent tumors and a lower total tumor burden (Fig. 1B, Supplementary Fig. S1A). In addition, expression of the proliferation marker KI67 was significantly decreased in the KPM2 lung tumors (Fig. 1C). Levels of the MSI2 homolog MSI1 were comparable in the KP and KPM2 lung tumors (Fig. 1C), indicating observed activity was specific to MSI2, rather than involving cross-regulation of Msi1. To define the mechanism underlying the reduced tumor growth phenotype upon Msi2 elimination, we established six murine lung tumor cell lines from KP and KPM2 mice: KP-1, KP-2, KP-3 and KPM2–1, KPM2–2, KPM2–3, respectively (Fig. 1D). Cell growth analysis of murine lung tumor cell lines using Cell Titer Blue (CTB) and clonogenic assays indicated significantly slower KPM2 cells growth in comparison with KP cells (Fig. 1E and F).

### Musashi-2 depletion leads to impaired of DNA damage response signaling and cell cycle in murine and human NSCLC

**Figure 2:**
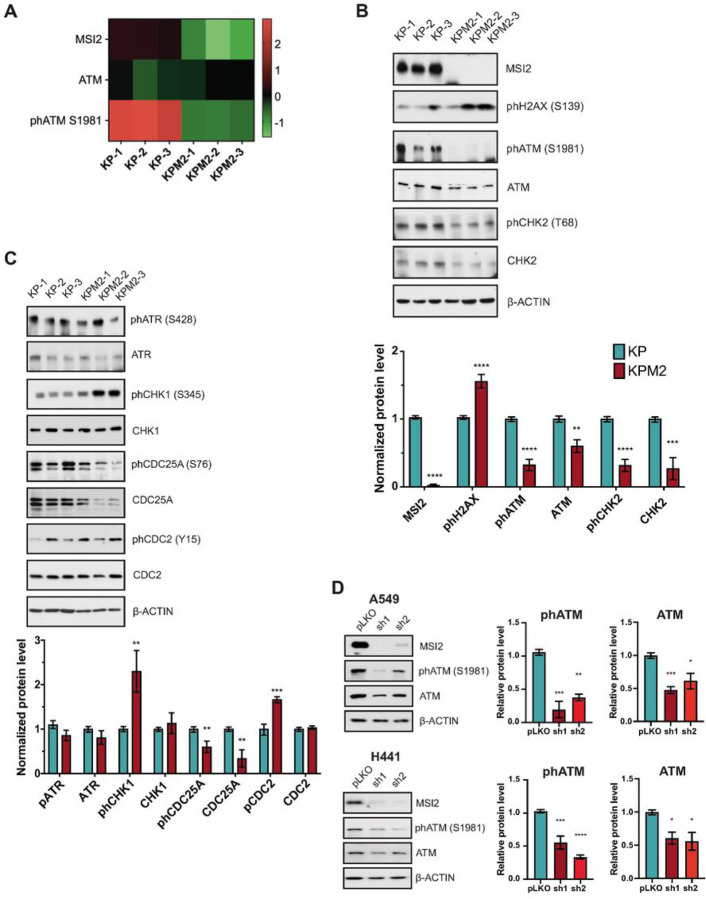
Figure legend not available with this version.

Since *Msi2* KO reduces lung tumor growth *in vitro* and *in vivo*, we performed Reverse Phase Protein Array (RPPA) analysis of the KP and KPM2 cell lines (Supplementary Table S8). RPPA data indicated that Msi2 depletion decreased expression of VEGFR2, SMAD3, and SOX2, which regulate tumor proliferation and invasion (Supplementary Table S8), confirming previously published studies on MSI2 function in other systems [22, 32, 40]. Unexpectedly, we found that loss of MSI2 caused significant reduction for total and auto-phosphorylated (S1981) ATM, suggesting a potential effect on DDR (Fig. 2A). Extended Western blot validation analysis showed that phospho-ATM, total ATM and its direct downstream target CHK2 and phospho-(T68) CHK2 are all significantly decreased with MSI2 knockout, despite increase in the level of phospho-(S139) H2AX (Fig. 2B, Supplementary Fig. S1B). Western blot analysis of additional relevant DDR signaling pathways (Fig. 2C, Supplementary Fig. S1B) indicated that phospho-(S345) CHK1 (an active form of this direct downstream target of ATR) is increased, while total and phospho- (S76) CDC25A protein levels were decreased and phospho-(Y15) CDC2 increased in MSI2 deficient KPM2 cells. Decreased CDC25A and as a result, increased phospho-CDC2 protein levels after activation of CHK1 is consistent with induction of checkpoints leading to cell cycle arrest [41–43].

To validate MSI2 regulation of ATM in human NSCLC, we used two human NSCLC adenocarcinoma cell lines (A549, H441) with MSI2 doxycycline-inducible knockdown (KD) (sh1, sh2), and evaluated phospho-ATM and total ATM levels after MSI2 inducible KD (Fig. 2D Supplementary Fig. S1C). Western blot analysis showed that MSI2 KD leads to significant decrease of both phospho-ATM and total ATM in human NSCLC cell lines. Thus, we conclude that MSI2 strongly supports ATM protein activity in both murine and human NSCLC.

**Figure 3:**
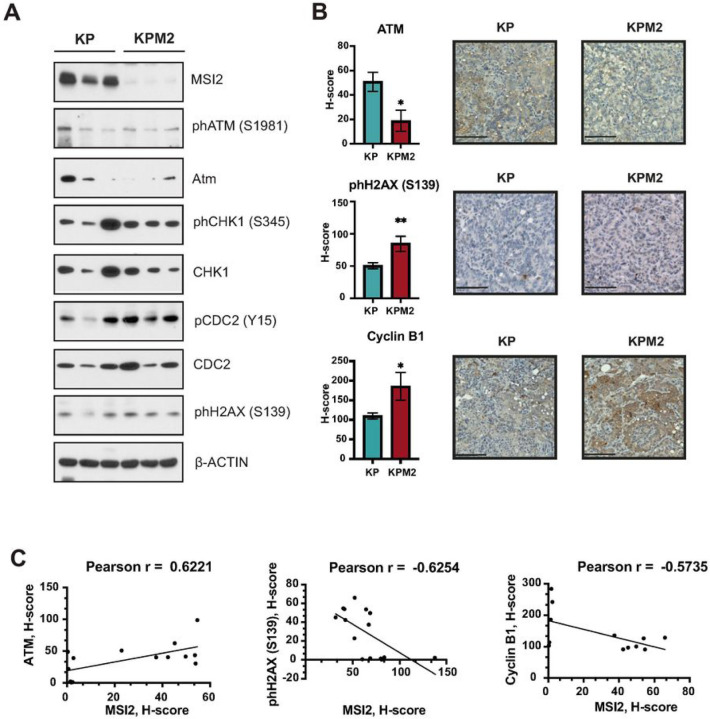
Figure legend not available with this version.

Next, we used murine lung tumor samples to evaluate the effect of Msi2 KO on DDR signaling in mouse lung tumors (Fig. 3). We performed Western blot analysis of tumor samples from 3 KP and 3 KPM2 mice (Fig. 3A). Our analysis showed a very significant decrease of ATM and phospho-(S1981) ATM, as well as increase of phospho-(S345) CHK1, phospho-(Y15) CDC2, and phospho-(S139) H2AX protein levels, consistent with data from murine and human lung cancer cell lines (Fig. 2A). ATR and CHK2 levels were undetectable in these primary lung tumors (data not shown). Next, we performed IHC analysis of murine tumor samples, which showed strong decrease of ATM and increase of phospho-H2AX (S139) with Cyclin B1 (Fig. 3B) and positive correlation of ATM with MSI2 (Pearson rank = 0.62), strong negative correlation of phospho-H2AX with MSI2 (Pearson rank = −0.625) and moderate negative correlation of Cyclin B1 with MSI2 (Pearson rank = −0.57) (Fig. 3C). Taken together, these data indicate that Musashi-2 supports DDR signaling and cell cycle progression in murine and human lung cancer.

### Musashi-2 directly regulates ATM protein levels in lung adenocarcinoma

**Figure 4:**
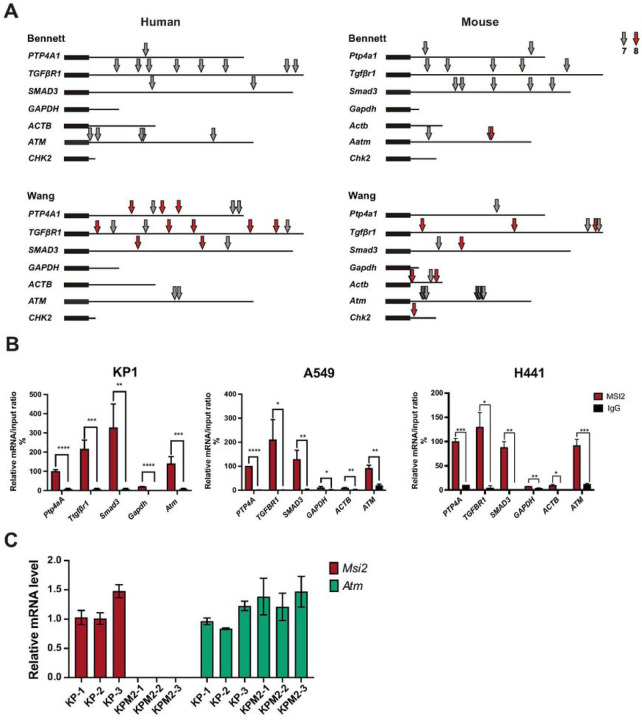
Figure legend not available with this version.

The above data suggests that the RNA-binding MSI2 protein may directly regulate translation of the *ATM* mRNA. Previous studies [23–25] have determined that MSI2 directly binds consensus sequences with a core UAG motif in the 3’-UTR of target mRNAs, and as a result regulates the stability and translation of target mRNAs. Based on that published data we performed *in silico* analysis and found that *ATM* mRNA has predicted binding sites for MSI2 binding (Fig. 4A, Supplementary Tables. S5, S6, S7). To confirm the *in silico* predictions, we performed RNA immunoprecipitation assays (RIP) with an MSI2 antibody coupled with RT-qPCR in 3 NSCLC cell lines: murine KP-1, and human A549, H441(Fig. 4A), using previously defined MSI2 target mRNAs (*PTP4A*, *TGFβR1*, *SMAD3*)[22, 33] as positive controls and ACTB and GAPDH as negative controls. Antibodies to MSI2 specifically immunoprecipitated the ATM mRNA as efficiently as they did the positive controls (Fig. 4B). In addition, we performed RTqPCR analysis of KP and KPM2 cell lines which showed that MSI2 KO has no effect on the ATM mRNA level (Fig. 4C). Taken together, we conclude that MSI2 directly regulates the ATM protein level in both murine and human lung adenocarcinoma via direct binding to *ATM* mRNAs.

### Musashi-2 depletion leads to sensitization of murine and human NSCLC to PARP inhibitor treatment

**Figure 5:**
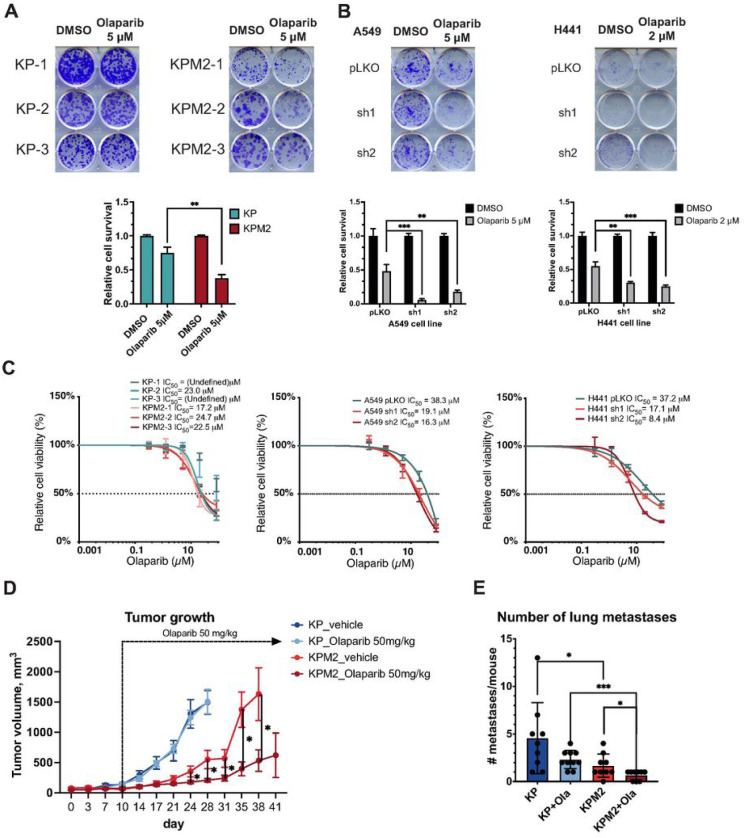
Figure legend not available with this version.

Several clinical trials have recently shown potential efficacy of PARP inhibitors for NSCLC, especially when combined with platinum-based chemotherapy or immunotherapy, although there is lack of clarity on specific biomarkers to select NSCLC patients that might benefit from this approach [13–16, 44]. In addition, recent publications show that ATM deficiency is associated with higher sensitivity to PARP inhibitor olaparib in NSCLC [18–20]. Taken together, we hypothesized that MSI2 depletion, which decreased ATM protein levels, might be associated with sensitization of NSCLC to olaparib treatment. To test this hypothesis, murine NSCLC KP and KPM2 cell lines were treated with olaparib (Fig. 5A and C, Supplementary Fig. S2A). Clonogenic and CTB viability analyses showed significant sensitization in cells with Msi2 KO to olaparib treatment. Also, western blot analysis of DDR signaling with olaparib treatment showed higher effect of this drug in KPM2 cells (Supplementary Fig. S3A). Levels of phospho-H2AX, phospho-CHK1, phospho-CDC2 are also higher post-olaparib treatment in KPM2 cells compared to KP cells. Olaparib treatment of human NSCLC cell lines also showed significant sensitization of cells with MSI2 inducible knockdown (Fig. 5B and C, Supplementary Fig. S2B).

We also used murine MSI2-expressing or deficient cell line models to evaluate the effect of MSI2 KO in the context of olaparib treatment *in vivo*. We used KP-1 and KPM2–2 cell lines for subcutaneous (SQ) injection. When tumor volumes reached 100 mm^3^, animals were dosed for 31 days with either olaparib (50 mg/kg, 5 days a week) or vehicle. Consistent with the *in vitro* experiments, MSI2 KO lead to significant tumor growth decrease and sensitization to olaparib treatments (Fig. 5D). Additionally, IHC analysis of SQ tumor xenografts showed slightly elevated phospho-(S139) H2AX level in KPM2 and KPM2 + olaparib groups relative to KP or KP + olaparib grops (Supplementary Fig. S3C). Moreover, H&E staining of murine lungs after 41 days of tumor growth shows that mice injected with MSI2 KO tumors have significant decrease in the number of lung metastases, which was further reduced by olaparib treatment (Fig. 5E, Supplementary Fig. S3D).

Finally, we performed in vitro treatments with platinum-based chemotherapy drug cisplatin. Murine lung tumor cell lines with MSI2 KO have significant sensitization to cisplatin treatment in comparison to KP cell lines (Supplementary Fig. S2C,D). Also, western blot analysis of DDR signaling with cisplatin treatment showed higher effect in KPM2 cells (Supplementary Fig. S3B). Levels of phospho-(S139) H2AX and phospho-(Y15) CDC2 are higher with cisplatin treatment in KPM2 cells than KP cells. In contrast to the murine results, MSI2 KD only weakly sensititized human NSCLC cell lines to cisplatin treatment, as measured by CTB viability and clonogenic assay (Supplementary Fig. S2E,F). Taken together, we conclude that Musashi-2 depletion leads to sensitization of both murine and human NSCLC to PARP inhibitor treatment and to sensitization to platinum-based chemotherapy only in murine and not in human NSCLC.

## Discussion

In the past decade, the Musashi proteins have been shown to influence tumorigenesis in numerous ways, enhancing stem cell pools, controlling the expression of oncogenes and other signaling pathways that promote growth and invasion. In this study, using new mouse model allowing inducible loss of MSI2 during NSCLC formation, we for the first time show that MSI2 regulates expression of DDR signaling proteins, in particular via control of ATM, resulting in persistent elevation of phospho-(S139) H2AX, a marker of DNA damage. Importantly, cells and tumors lacking MSI2 were sensitized to PARP1 inhibition, compatible with a deficiency in DNA repair and suggesting a potential synthetic lethal strategy to improve the use of PARP1 inhibitors in the clinic.

A large body of literature indicates that DDR pathways promote cancer cell survival and therapeutic resistance[45]. A number of studies have assessed the value of DDR pathway alterations in predicting NSCLC response to platinum therapy with equivocal results, and DDR alterations are not currently reliable as predictive biomarkers for chemosensitivity [15]. More recently, clinical trials of protein-targeted PARP inhibitors have demonstrated that these may benefit subsets of lung cancer patients [15, 46]. Furthermore, in a cohort of unselected patients with advanced NSCLC patients, olaparib maintenance led to improvements in survival, although it was not significant [47]. For these targeted inhibitors, useful predictive biomarkers of response to these drugs in NSCLC include HRD scores, mutations in specific DNA repair pathway genes (BRCA2 and others), and gene expression profile [15]. Several studies have shown that deletion of ATM in murine models of NSCLC, or ATM depletion in human NSCLC cell lines, is sensitizing to PARP inhibitors [19, 48]. ATM depletion also sensitized NSCLC cells to ATR inhibitor, which was further enhanced by combining ATM depletion with cisplatin or gemcitabine [18]. The recent development of new inhibitors of ATM (AZD0156) and ATR (BAY1895344) [49, 50], provides potentially valuable new tools; it will be of interest to evaluate whether loss of MSI2 influences their efficacy in vitro and *in vivo*.

The Musashi proteins have been most studied as oncoproteins, and as stemness factors in normal and cancer stem cells [51, 52]. Relevant to this role, our data showed that lung cancer cell lines derived from KPM2 mice demonstrate decreased proliferation and clonogenic survival compared to those from KP mice, while RPPA analysis indicated that Msi2 depletion decreased pro-oncogenic factors VEGFR2, SMAD3, and stem cell transcription factor SOX2. Interestingly, cancer stem cells in NSCLC, glioma and other tumor types are known to have increased DNA repair capacity [53–55]. Our data indicate that elevated DDR in stem cells may be directly related to MSI2 support of DDR proteins, with ATM one direct MSI2 target.

To date, there has been no investigation of MSI2 as a regulator of DDR. However, the Drosophila Musashi homolog has been implicated in control of DDR, with Musashi expression in Drosophila intestinal stem cells attenuating radiation-induced reduction in intestinal permeability and promoting proliferation of these cells [56]. Further, knockdown of the MSI2 paralog MSI1 in glioblastoma led to a decrease in cell survival and an increase in DNA damage compared to control cells following treatment with ionizing radiation, in part via decreased expression of DNA–protein kinase catalytic subunit (DNA-PKc), which is a direct MSI1 target [57, 58]). Our study has now established MSI2 as another critical regulator of DNA repair in cancer. Whether MSI2 also influences DDR and levels of phospho-H2AX through control of DNA-PKc [59] remains to be established.

Because of the absence of easily druggable domains in noncatalytic RNA-binding proteins, it has been challenging to develop effective small molecule inhibitors targeting Musashi proteins. However, a neural growth factor (NGF) inhibitor developed by Roche, Ro 08–2750[60, 61], has recently been found to inhibit MSI2/MSI2 both in vitro and in vivo [62]. In addition, we have recently taken part in development of a first-in-class Musashi inhibitor, which effectively interferes with RNA Recognition Motif 1 (RRM1) binding of Musashi proteins to its targets [63]. Further refinement of these inhibitors, including generation of more potent compounds or degraders of Musashi is warranted, and may ultimately yield valuable new lung cancer therapies.
